# Incidence, Temporal Trends, and Departmental Distribution of Reported Needle-Stick Injuries Among Healthcare Workers at Hera General Hospital, Saudi Arabia (2022–2024): A Retrospective Clinical Review

**DOI:** 10.7759/cureus.100221

**Published:** 2025-12-27

**Authors:** Heba Dosh, Bashaer O Abdu, Nada A Alreheily, Mohammad K Alharazi, Abdulaziz A Joharji

**Affiliations:** 1 Preventive Medicine, Ministry of Health, Makkah, SAU; 2 Preventive Medicine, Hera General Hospital, Makkah, SAU; 3 Preventive Medicine Nursing, Hera General Hospital, Makkah, SAU; 4 Public Health and Preventive Medicine, Ministry of Health, Makkah, SAU; 5 General Practice, Alnoor Hospital, Makkah, SAU

**Keywords:** healthcare workers, hospital surveillance, infection control, needle-stick injuries, occupational exposure, patient safety, reporting system, saudi arabia, temporal trends, underreporting

## Abstract

Background

Needle-stick injuries (NSIs) pose a critical occupational risk for healthcare workers, with profound implications for health, safety, and patient care. The aim of this study was to evaluate the incidence, temporal trends, and departmental distribution of reported needle-stick injuries among healthcare workers at Hera General Hospital from 2022 to 2024. The primary objective was to determine overall and annual injury rates, while secondary objectives included assessing variations by year, department, cause, and month, and identifying potential reporting gaps relevant to occupational safety.

Methods

This retrospective observational study analyzed data from 160,092 healthcare worker records. Data were collected from occupational medical health clinic notes and supplemented with clarification on phone calls when required. The rates of NSIs were calculated per 1,000 records. Data were analyzed using SPSS v29.0 (IBM Corp., Armonk, USA). Numerical variables were expressed as mean ± SD, and categorical variables as frequencies and percentages. Normality was assessed using the Kolmogorov-Smirnov test. Associations were evaluated with the Kruskal-Wallis test, with p < 0.05 considered significant.

Results

Among 160,092 records, 114 NSIs were reported with an overall rate of 0.709 per 1,000 records. Annual rates were 0.675 in 2022, 0.638 in 2023, and 0.815 in 2024, showing no significant difference (p = 0.593). Most injuries were accidental (97 cases, 85.1%), with the operating room (26 cases, 22.8%) and emergency department (25 cases, 21.9%) recording the highest prevalence. Temporal peaks were observed in February 2024 (N = 16; 1.916) and October across all years (N = 44; 1.122). Departments like pediatric intensive care (0 cases) and central sterile services (1 case) reported minimal to no cases. Analysis revealed no significant differences in monthly rates across the years (p = 0.349).

Conclusion

Needle-stick injuries s are predominantly accidental, with notable temporal and departmental variation. Focused training, strict adherence to safety protocols, and enhanced reporting systems are essential to reducing NSIs and improving healthcare worker safety at Hera General Hospital. Focused training, strict adherence to safety protocols, and enhanced reporting systems are essential to reducing NSIs and improving healthcare worker safety at Hera General Hospital.

## Introduction

Needle-stick injuries (NSIs) are one of the most common occupational hazards faced by healthcare workers, presenting a persistent challenge within healthcare settings globally [1]. These injuries not only pose a significant risk of transmitting bloodborne pathogens, including hepatitis B virus, hepatitis C virus, and human immunodeficiency virus, but also contribute to considerable emotional and economic burden for the affected individuals and the healthcare system at large [2]. In the Makkah region, Saudi Arabia, particularly at institutions like Hera General Hospital, understanding the incidence and reporting patterns of NSIs, identifying contributing factors, and implementing effective preventive measures are critical components of enhancing workplace safety and health outcomes.

Globally, the prevalence of NSIs is alarmingly high, with millions of healthcare workers affected each year. The global prevalence of NSIs among healthcare workers is 44.5%, with regional variations. The highest rates are in the Eastern Mediterranean region (53.5%) and South-East Asia (58.2%), with lower rates in Western countries [3]. Moreover, there are also significant differences in the prevalence of NSI by gender, with 21.4% for female adolescents and 13.7% for male adolescents [4]. These injuries are especially prevalent in environments where the pace of work is fast, the procedures involving sharps are frequent, and the adherence to safety protocols is compromised by either lack of resources or training [5]. Current medical literature indicates that approximately 28.5% of needle-stick injuries (NSIs) go unreported, which complicates efforts to ascertain their true prevalence and undermines initiatives aimed at improving healthcare worker safety [6]. In the Middle East, and Saudi Arabia in particular, studies suggest that NSIs are a significant occupational risk for healthcare workers [7]. According to a 2022 study, the one-year incidence of at least one NSI event among healthcare workers is projected to be 22.2% [7]. Additionally, a 2024 study discovered that the average incidence rate per 100 occupied beds was 25.43 [8]. However, comprehensive longitudinal data based on occupational health records specific to local contexts like Makkah is often lacking.

The contributing factors to NSIs are multifaceted, involving individual, procedural, and systemic issues. Individual factors include the skill level of the healthcare worker, their experience, fatigue, and even psychological stress and fatigue, which can affect concentration and procedural compliance [9]. Procedural factors involve the types of medical devices used, the frequency of procedures requiring sharps, and the urgency and complexity of these procedures [10]. Systemic factors encompass the healthcare setting’s policies on safety, availability, and use of protective devices, training on their proper use, and the cultural attitude towards safety [3].

Preventive strategies for NSIs have been extensively studied and generally focus on three main areas: engineering controls, administrative controls, and personal protective equipment (PPE). Engineering controls involve the use of safer medical devices, such as needles with safety features designed to prevent injury [11]. Administrative controls include training programs, clear procedural protocols, and a supportive environment that encourages the reporting and discussion of safety issues [12]. Personal protective equipment must be provided and used appropriately, but its effectiveness also depends on consistent adherence to safety practices [11].

Despite these known measures, the implementations of effective prevention strategies in healthcare setting varies widely. Barriers to effective implementations include a lack of resources, inadequate training, resistance to change in established practice, and underreporting of incidents, which diminishes the perceived urgency to address these risks [13, 14].

According to guidelines in Saudi Arabia, healthcare workers (HCWs) exposed to needle stick injuries (NSIs) or bodily fluids should promptly wash the area, report the incident, identify and test the source patient, and seek medical evaluation for prophylaxis and follow-up testing for bloodborne pathogens like HIV, Hepatitis B, and Hepatitis C [15].

The significance of these injuries extends beyond the immediate health implications; they also impose economic burdens due to the cost of post-exposure prophylaxis, potential long-term healthcare if transmission of infection occurs, and the psychological impact on the healthcare workers, which can affect job satisfaction, performance, and retention [16].

Training on universal precautions, safe injection techniques, appropriate sharps disposal, the use of safety-engineered devices, and Hepatitis B immunization are all standard procedures in Saudi hospitals to prevent needle-stick accidents. Changes in NSI incidence, staff knowledge and adherence surveys, and audits of safety procedure compliance, which have demonstrated lower injury rates and better reporting, are typically used to gauge effectiveness [7, 17].

The rationale for studying reported NSIs among healthcare workers across the Kingdom of Saudi Arabia stems from the need to address significant occupational health risks. NSIs pose transmission risks for serious infections like HIV and hepatitis, varying significantly by region due to differences in safety practices and protocol adherence. Understanding these variations is crucial for tailoring effective safety policies, allocating resources appropriately, and enhancing training programs.

This study aimed to evaluate the incidence, temporal trends, and departmental distribution of reported needle-stick injuries among medical staff at Hera General Hospital in Makkah, Saudi Arabia, between 2022 and 2024. The primary objective was to determine the overall and annual incidence rates of reported needle-stick injuries based on occupational health clinic records. Comparing injury rates over the course of the three years, analyzing their distribution by hospital department, identifying reported causes of injuries, looking at monthly and seasonal variations, and identifying potential reporting gaps that may influence occupational safety surveillance and prevention strategies.

## Materials and methods

Study design and location

This is a retrospective observational study conducted at Hera General Hospital, Makkah, Saudi Arabia, that covered the period from 2022 to 2024. Hera General Hospital was chosen as a major tertiary referral hospital in Makkah that serves a high-volume and seasonally fluctuating population (including Hajj and Ramadan), making it a relevant setting for assessing NSI burden.

Study population

All 160,092 healthcare worker records that were turned in to the Occupational Medical Health Clinic during the study period were examined. Prevalence was determined per record rather than per healthcare worker because each record can reflect a different visit or several reports from the same person. Secondary data from Hera General Hospital's Occupational Medical Health Clinic was used to determine the prevalence of NSIs. During the study period, all officially reported NSI cases and all healthcare worker data (n = 160,092) were included. The proportion of records documenting at least one reported NSI in relation to the total number of occupational health clinic records examined was referred to as prevalence. The estimated prevalence reflects reported NSIs documented in occupational health clinic records and may underestimate the true occurrence due to underreporting. Since every entry had the information needed to identify reported needlestick injuries, no records were excluded.

The study was a census of available records rather than a sample since it included all healthcare worker records that were submitted to the occupational medical health clinic at Hera General Hospital between 2022 and 2024. Since the whole population of interest was included in the analysis, no sampling approach was used.

The dataset includes demographic information, job-related parameters, exposure history, clinical assessment, and follow-up documentation. Exclusion criteria included incomplete reports, duplicated entries, and non-healthcare worker exposures.

Study procedures

Data were gathered retrospectively from the Occupational Medical Health Clinic electronic records. The standardized occupational NSI reporting forms and the electronic Occupational Medical Health Clinic database were the data sources. Variables from the study were used to evaluate contributing factors for needlestick injuries. These included the conditions under which the injury occurred, the clinical department or place of employment, and temporal elements like the accident's year and month. The analysis looked at changes in NSI rates over time and between departments. Due to the secondary data source's limitations, individual behavioral, procedural, and device-related aspects were not assessed. In order to standardize comparison, NSI rates were computed per 1,000 records.

Injuries that happen unintentionally while providing standard medical care or handling sharp objects, such as needlesticks during surgery or injection administration, are referred to as accidental pricks. Generally speaking, "pricks caused by mistake" refers to occurrences that arise from mistakes in procedure, inattention, or normal safety protocol violations, like recapping a needle or inappropriate disposal. In accordance with established standards used in occupational health monitoring, classification was based on the description given in each event report by the healthcare worker at the time of reporting [18,19,20].

Inadvertent activities that result in occupational exposure, such as unintentional needle pricks or inappropriate handling of sharps, are referred to as unintentional mistakes or blunders. Neglect is defined as a failure to adhere to basic measures or established safety protocols, such as recapping needles or improperly disposing of sharps, which raise the risk of damage [7,18,21]. Both groups are important targets for occupational safety initiatives since they are thought to be preventable.

Ethical considerations

An ethical approval for the study was obtained from the research ethics committee of the Ministry of Health, General Administration for Research & Studies, Saudi Arabia, with the ethical clearance number H-02-K-076-1025-1467. No study activities were started until the IRB approval was obtained.

Statistical analysis plan

Data were collected in a Microsoft Excel (Microsoft Corporation, Redmond, USA) sheet and then transferred to SPSS software, version 29.0 (IBM Corp., Armonk, USA), for statistical analysis. Descriptive statistics were used to summarize and report the variables. Numerical data were presented as mean ± standard deviation (SD), while categorical variables were expressed as frequencies and percentages. The Kolmogorov-Smirnov test was used to assess the normality of numerical data. Since the data were not normally distributed, the Kruskal-Wallis test was used to evaluate associations between variables. A p-value of less than 0.05 was considered statistically significant.

## Results

**Figure 1:**
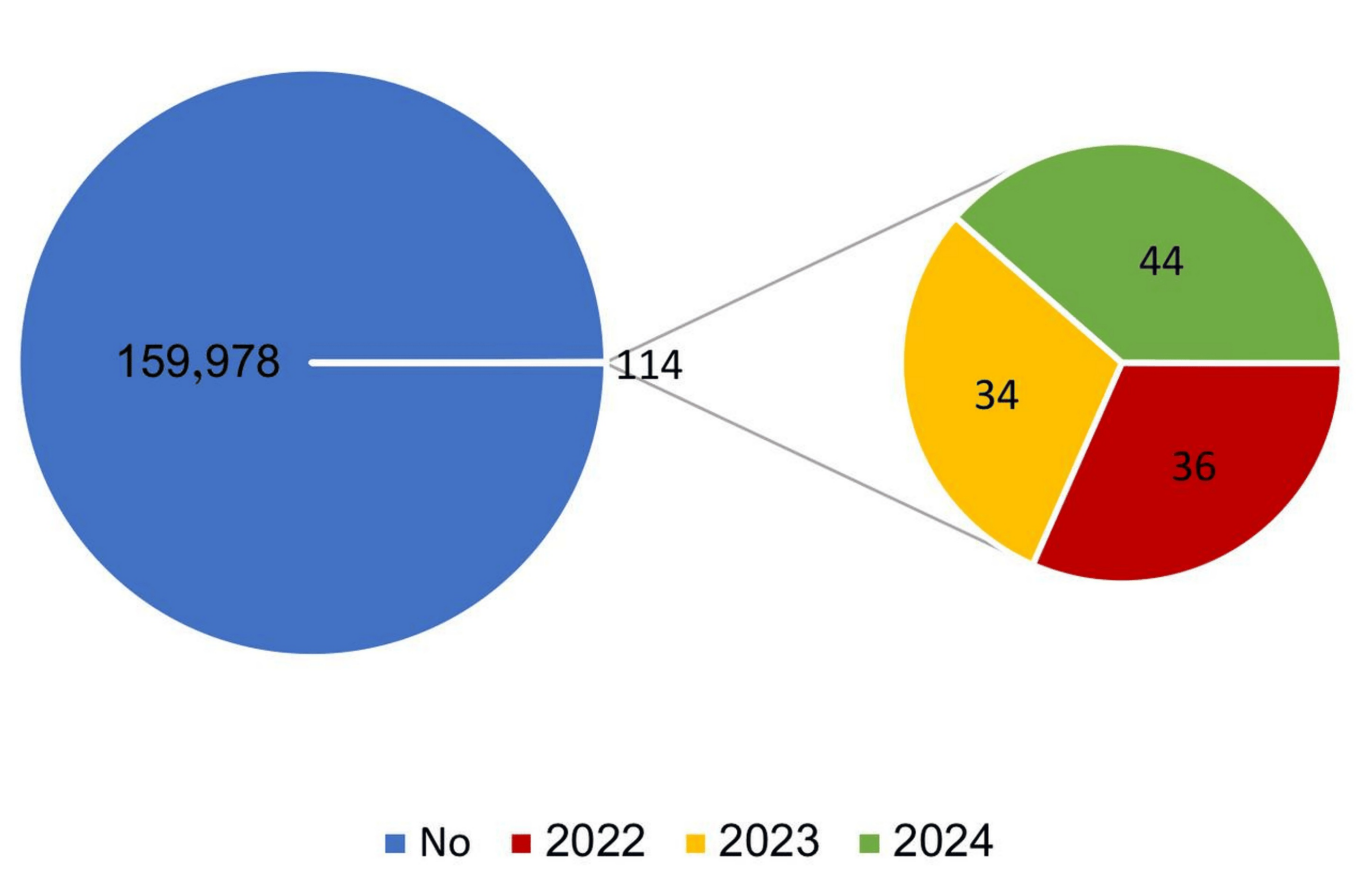
Prevalence of needle-stick injuries among healthcare workers in different departments

A total of 160,092 occupational health clinic records from Hera General Hospital in Makkah, Saudi Arabia, between 2022 and 2024 were included in the current study. The overall average rate of needle-stick injuries throughout the three years was found to be 0.709 per 1,000 records (114/159,978; 0.071%). The rate of needle stick injury in 2022 was 0.675 per 1,000 cases (36/52,600; N = 36, 0.068%), 0.638 per 1,000 records (34/53,251; N = 34, 0.064%) in 2023, and 0.815 per 1,000 records (44/54,241; N = 44, 0.081%) in 2024. There was no statistically significant difference in needle-stick injury rates across the three years (P = 0.593) (Figure 1).

Among the reported cases of needle-stick injuries (n = 114), the majority of the cases were caused by accidental pricks (n = 97, 85.1%) while 17 (14.9%) were caused by mistakes or negligence. Among departments, the operating room was the most reported department (n = 26, 22.8%), followed by the emergency department (n = 25, 21.9%), auxiliary services (n = 21, 18.4%), obstetrics and gynecology (n = 10, 8.8%), and internal medicine department (n = 7, 6.1%). The lowest rates of needle-stick injuries were observed in pediatric intensive care unit (n=0, 0.0%), central sterile services department (n=1, 0.9%), and neonatal intensive care unit (n=2, 1.8%) (Table 1).

**Table 1:** : Cause category and department of needle-stick injuries (n = 114)

Variable:	Categories:	N (%)
Cause	Mistake/ negligence	17 (14.9)
	Accidentally pricked	97 (85.1)
Department	Laboratory	5 (4.4)
	Emergency department (ED)	25 (21.9)
	Intensive care unit (ICU)	4 (3.5)
	Pediatric intensive care unit (PICU)	0 (0)
	Neonatal intensive care unit (NICU)	2 (1.8)
	Internal medicine department (IMD)	7 (6.1)
	Surgery (SGY)	6 (5.3)
	Operating room (OR)	26 (22.8)
	Obstetrics and gynecology (OBG)	10 (8.8)
	Central sterile services department (CSSD)	1 (0.9)
	Auxilliary services	21 (18.4)
	Outpatient department (OPD)	6 (5.3)

Monthly needle-stick injury rates per 1,000 records were calculated across the three years. Showing that in 2022, January had the highest rate of needle-stick injuries (1.235 per 1,000; approximately N = 65, 1.2%), followed by October (1.099 per 1,000; N ≈ 58, 1.1%). In 2023, October had the highest rate (1.391 per 1,000; N ≈ 74, 1.4%) followed closely by November (1.345 per 1,000; N ≈ 72, 1.3%), while in 2024, February recorded the highest rate (1.916 per 1,000; N ≈ 104, 1.9%) followed by June (1.433 per 1,000; N ≈ 78, 1.4%). The rate of needle-stick injury in 2022 was 0.675 per 1,000 cases (36/52,600; 0.07%), 0.638 per 1,000 cases (34/53,251; 0.06%) in 2023, and 0.815 per 1,000 cases (44/54,241; 0.08%) in 2024. There was no significant difference among the 3 years regarding the rate of needle-stick injuries (P = 0.593) (Table 2).

**Table 2:** Needle-stick injuries rates by month for the years 2022-2024 ^*^Kruskal-Wallis test

Month	Rate of needle-stick injuries per 1,000 records		
	2022	2023	2024
January	1.235	0.672	1.183
February	1.025	0.236	1.916
March	0.226	0.223	0.449
April	0.497	0.894	0.720
May	0.459	0.429	0.455
June	0.984	0.225	1.433
July	1.064	1.052	0.219
August	0.440	0.486	0.487
September	0.413	0.486	0.450
October	1.099	1.391	0.875
November	0.224	1.345	0.439
December	0.429	0.212	1.157
Overall rate	0.675	0.638	0.815
p-value*	0.593		

**Figure 2:**
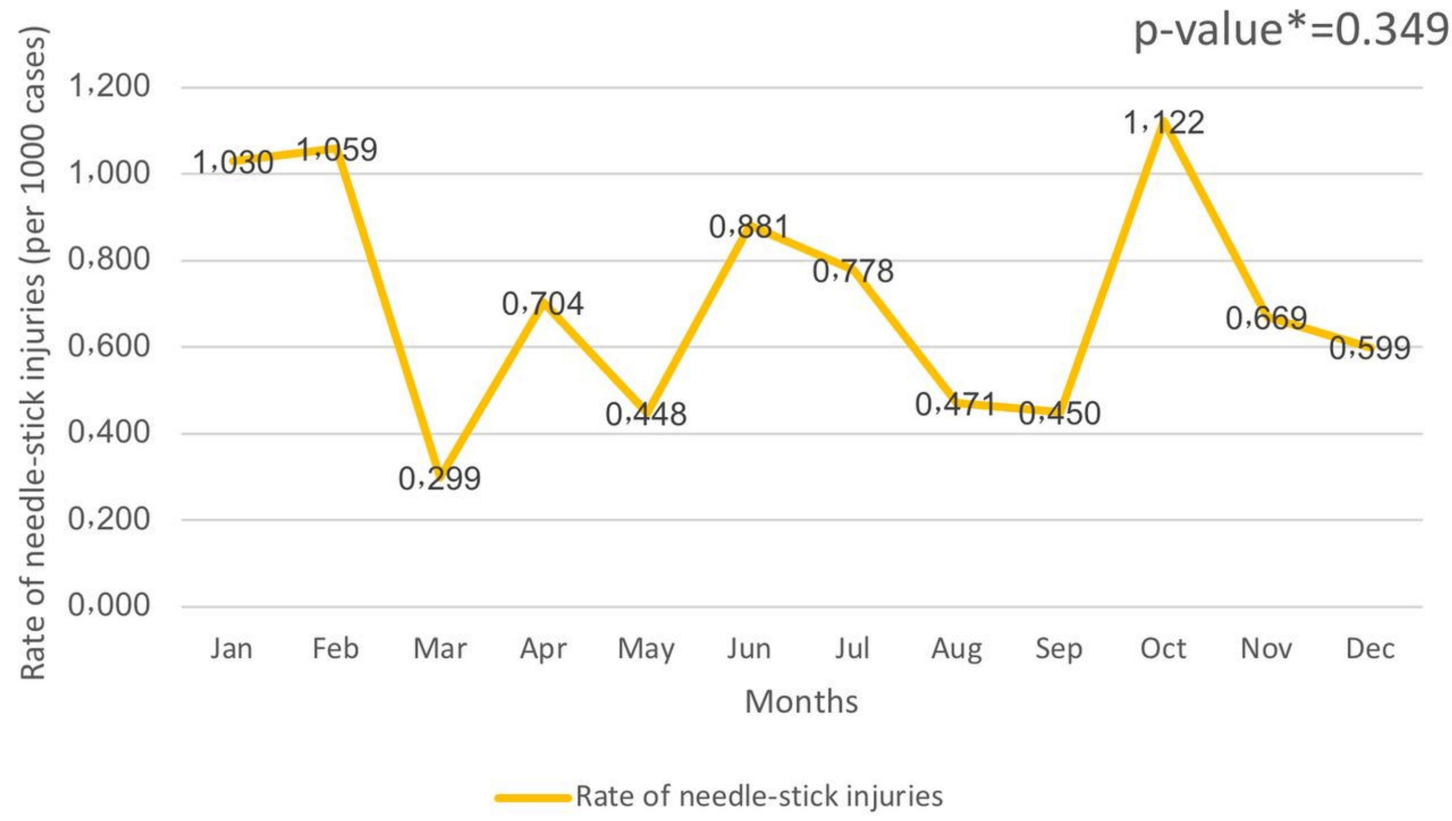
Needle-stick injuries rates according to month *Kruskal-Wallis test

Throughout the three years, the average rate of needle stick injury for each month was calculated. Over the three years, October recorded the highest rate of needle-stick injuries (1.122 per 1,000; N ≈ 60, 1.1%), followed by February (1.059 per 1,000; N ≈ 57, 1.0%) and January (1.030 per 1,000; N ≈ 56, 1.0%), while May (0.448 per 1,000; N ≈ 24, 0.4%), September (0.450 per 1,000; N ≈ 24, 0.4%), and March recorded the lowest rate (0.229 per 1,000; N ≈ 12, 0.2%). The results also showed that there was no statistically significant difference in needle-stick rates between months (P = 0.349) (Figure 2).

## Discussion

NSIs are a significant occupational hazard for healthcare workers, posing risks of transmitting blood-borne pathogens such as hepatitis B, hepatitis C, and HIV [10]. Globally, approximately 3 million healthcare workers experience sharps injuries annually, with substantial underreporting complicating accurate assessments. This study aimed to assess the incidence rates of reported needle-stick injuries based on occupational health clinic records at Hera General Hospital in Makkah, Saudi Arabia, from 2022 to 2024. The overall average rate of NSIs was 0.709 per 1,000 records, with annual rates of 0.675, 0.638, and 0.815 per 1,000 records for 2022, 2023, and 2024, respectively. There was no statistical difference between the three years (P = 0.593). The reported NSI rates at Hera General Hospital are considerably lower than global estimates reported in the literature. A meta-analysis by Bouya et al. (2020) revealed a one-year global pooled incidence of NSIs among HCWs at 44.5%, with the South-East Asia region recording the highest incidence at 58.2% [3]. Similarly, a cross-sectional survey conducted in Saudi Arabia found that the one-year NSI incidence rate was 22.2% among healthcare workers, and 36% among physicians, and 34.8% among nurses [7].

A higher prevalence of NSIs is also possible based on the healthcare facility and the area in question. For instance, a systematic review by Bevan et al. (2023) revealed that the pooled prevalence rate of NSIs in operating theatres ranged from as high as 41.5% in some settings, capturing the high-risk nature of these environments [22]. A study conducted in Riyadh, Saudi Arabia, by Alqahtani et al (2021) revealed an incidence rate of 124 per 1,000 employees; however, it established that there was high under-reporting [23]. The lower NSI incidence in Hera General Hospital may be due to the proper enforcement of the safety measures; another explanation could be the retrospective study design, which may underestimate the outcome. Another reason could be the training of the personnel and the provision of safety-engineered devices. Nevertheless, it is necessary to take into account the phenomenon of underreporting, which is an acute problem in the NSI surveillance. Several investigations have shown that the majority of the NSI events go unreported by healthcare workers, with reasons such as low perceived risk or complicated reporting systems [21, 24].

The slight increase in reported NSI rates in 2024, although not statistically significant, warrants attention. Some of the possible causes of such an increase could include: a large number of patients, changes in personnel, or non-compliance with precautions. Similar results have been documented in other Saudi Arabian research, despite the fact that our study lacked direct data on patient load, personnel changes, or adherence to safety protocols. Higher patient volumes, staff composition changes, and noncompliance with infection control procedures have all been linked to an increase in needlestick injuries among healthcare professionals, according to prior studies [25,26]. Therefore, even though the increase in our study was not statistically significant, these reasons are reasonable explanations for the observed increases. Therefore, the regular assessment and improvement of the prevention strategies are vital for sustaining and improving the NSI rates [6].

Of the reported needle-stick injuries identified in this study, the majority (85.1%) were accidental, occurring despite adherence to standard procedures, while 14.9% were associated with lapses in protocol adherence or procedural errors. This is in concordance with the global statistics, which pointed out that the majority of the cases are caused by accidental piercings among healthcare workers. In their study, Bouya et al. identified recapping of needles and disposal of used sharps as the main causes of NSIs and stressed the importance of training and compliance with safety guidelines [3]. A study conducted in Dammam Medical Complex, Saudi Arabia, also showed that accidental handling was the commonest cause of NSIs; this could be due to ignorance, fatigue, and working pressure from handling many patients [10]. The nature of NSIs falling under the departmental distribution showed that the operating room had the highest frequency (22.8%), followed by the emergency (21.9%) and auxiliary services (18.4%). This pattern is in line with the general global trends where surgical and emergency wards are identified as the most prone to occurrences because of the kind of procedures done and the pressure environment [27]. For instance, a systematic review by Abdelmalik et al. (2023) also found surgical and emergency departments to be the most frequent sources of NSIs globally. These environments present the healthcare workers with sharp objects, limited time, and many patients, which enhances the risks of injuries [28].

Auxiliary services, which constitute 18.4% of the total injuries in this study, are a subgroup of healthcare workers who may be exposed to NSIs but are often not considered as such. Employees in this category are at high risk of being stuck by a sharp from improperly discarded sharps during tasks like waste disposal and instrument sterilization. This is in agreement with the previous studies from the developing countries, where inadequate sharps disposal facilities have been highlighted to have a great impact on the non-clinical workers’ injuries [29]. Departments with low reported NSI counts, including the pediatric ICU (PICU), NICU, and Central Sterile Services Department (CSSD), may reflect effective safety practices; however, potential underreporting cannot be excluded. This may indicate good safety practices, but since underreporting is a known bias in NSI surveillance, this should be taken with caution. Literature has established that underreporting of NSIs is prevalent, with one study showing that as much as 70% of NSIs are not reported across the globe due to factors such as fear of blame or lack of understanding that reporting is vital [30]. Furthermore, in a systematic review and meta-analysis by Behzadmehr et al. (2023), the overall proportion of unreported NSIs was 59.9%, and the primary reason for this was the negligible concern regarding the injury [31].

Analysis of reported needle-stick injury rates from 2022 to 2024 revealed monthly fluctuations, with peaks in specific months each year. For instance, January 2022 had the highest rate (1.235 per 1,000 cases), October 2023 peaked at 1.391, and February 2024 recorded a rate of 1.916. Despite these monthly variations, the overall annual rates did not differ significantly (P = 0.593). These findings align with recent studies highlighting temporal patterns in NSI occurrences. A descriptive-analytical study by Mousavi et al. (2023) emphasized that factors, such as increased workload during specific periods, can elevate NSI risks among healthcare workers [32]. The observed monthly variations in NSI rates may be influenced by several factors, including seasonal workload fluctuations, staffing levels, and varying patient volumes. For instance, higher NSI rates in January and February could correlate with increased patient admissions during winter months, potentially leading to heightened workloads and fatigue among healthcare workers. Similarly, peaks in October and November might be associated with specific hospital activities or staffing patterns during these months [32].

The lack of significant annual variation in our study aligns with global data indicating that, without targeted interventions, NSI rates tend to remain stable over time [33]. Underreporting remains a significant issue in NSI data collection. There may have been underreporting of needlestick injuries (NSIs) at the research site. The actual incidence of NSIs may be underestimated because this investigation relied on retrospectively reported cases that were included in the hospital monitoring system. In Saudi healthcare settings, underreporting of NSIs has been extensively documented. According to earlier research conducted in Saudi Arabia, a significant percentage of healthcare workers fail to report non-communicable diseases (NSIs) for a variety of reasons, including perceived low risk of infection, lack of time during hectic shifts, fear of disciplinary action or blame, unfamiliarity with reporting procedures, and complicated reporting systems [7,18]. The comparatively low observed NSI rates and the lack of statistically significant variations over years and months may be partially explained by this possible underreporting, which is a significant study constraint.

The 2023 Chinese survey highlighted that more experienced healthcare workers exhibited higher rates of both NSIs and underreporting, suggesting that familiarity with procedures might lead to complacency in reporting protocols [6]. Addressing these barriers through education, streamlined reporting systems, and fostering a culture of safety is crucial for accurate data collection and prevention efforts [31].

The higher NSI rates observed in October, February, and January at Hera General Hospital may be associated with increased healthcare activities during these months. In Makkah, significant events such as Ramadan and the Hajj pilgrimage, which often occur around these months, lead to a surge in patient numbers, thereby increasing the workload for healthcare workers [28]. Elevated workloads have been identified as a contributing factor to NSIs, as they can lead to fatigue and lapses in adherence to safety protocols [34]. The lower NSI rates in March, May, and September could be related to relatively lower workloads or effective staff distribution during these months. Additionally, some studies emphasize the role of staff training and compliance with safety protocols during off-peak months, which may contribute to reduced rates of NSIs [34].

NSIs are reported via incident forms to employee health or infection control at the study site, and appropriate follow-up is started. Due to unclear protocols and insufficient knowledge, underreporting is frequent, and reporting is uneven. To lower injuries and guarantee prompt post-exposure management, improvements include staff education on NSIs and reporting, streamlined processes, a single national policy, and continuous training on safe practices and safety equipment [7,18]. Implementing targeted interventions during peak months, notably October, February, and January, is essential to mitigate the increased risk associated with higher patient volumes and intensified workloads. Additionally, addressing the pervasive issue of underreporting through streamlined reporting systems and fostering a culture of safety is imperative for accurate surveillance and effective prevention strategies. At Hera General Hospital, a focused strategy to lower needle-stick injuries (NSIs) is advised, especially during busy months when rates are at their greatest. Increased staff training on safe sharps handling and disposal, requiring the use of safety-engineered equipment in high-risk departments, and modifying staffing schedules to control workload and lower fatigue-related errors are some specific treatments. To increase compliance and avoid occupational exposures, it is also advised to actively monitor and promptly report NSIs, as well as to conduct frequent audits and update safety procedures. By putting these strategies into practice, the research site's human and system-related risk factors that contribute to NSIs may be addressed. By focusing on these areas, healthcare facilities can enhance occupational safety, reduce NSI incidence, and safeguard healthcare workers from potential blood-borne pathogen exposures [6, 31].

This study has a number of noteworthy strengths, even if it is based on secondary data. First of all, it is the first hospital-based analysis of needle-stick injuries (NSIs) in the Makkah region, offering distinctive, area-specific data on the incidence, departmental distribution, and temporal trends of NSIs in a high-volume tertiary hospital. Second, a thorough assessment of reported NSIs across all hospital departments and time periods is made possible by the study's extensive dataset of 160,092 healthcare worker records submitted over a three-year period. Third, using standardized occupational health records reduces variability in case identification and guarantees consistency in data reporting. Fourth, the study used non-parametric techniques (Kruskal-Wallis test) and systematic statistical analysis, such as normality testing, to look at patterns over time and between departments. Lastly, the study adds to the body of evidence supporting improvements in occupational safety in Saudi Arabian healthcare settings by highlighting high-risk times and departments that might profit from focused preventive interventions. This information is useful for hospital administrators and legislators.

Limitations

The study has several limitations. First, only needlestick injuries that were officially reported to the Occupational Medical Health Clinic were included in this retrospective research based on secondary data, which may have led to an underestimation of the actual prevalence. A thorough examination of the conditions and risk factors connected to NSIs may be hampered by incomplete or missing information in medical records. Second, Multiple entries per individual may have resulted in an overestimation or underestimation of NSI rates per record, and the total number of records may not accurately represent the number of individual healthcare workers. When analyzing the results, this limitation should be taken into account. Third, this retrospective study relied on reported NSI cases, likely underestimating the true incidence. Contributing factors such as protocol adherence, staff training, and workload were not directly assessed, and no interventions were implemented to evaluate their effect. Also, relying on secondary occupational health records that did not include demographic information or details limited the analysis of individual-level risk factors.

Fourth, as this is a single-center study, the findings may not be generalizable to other healthcare settings with different patient loads, safety protocols, or cultural practices. Additionally, the study relies on retrospective data collection and self-reporting from occupational health clinic notes, which may be subject to recall bias. Fifth, the examination of factors impacting NSIs and underreporting was limited since this retrospective study employed secondary data and lacked information on safety procedures, personnel training, experience, and use of safety devices. The results may not be generalizable because they come from a single hospital. In addition, the accuracy and perception of temporal patterns may also be impacted by irregularities in monthly NSI counts. Lastly, the absence of imaging and detailed procedural documentation may limit the comprehensiveness of the reported findings.

## Conclusions

The findings highlight that NSIs are an ongoing occupational hazard, with higher rates in high-demand months and departments like the operating room and emergency department. Preventive measures, including targeted education, adherence to safety protocols, and robust reporting systems, are essential. Addressing peak periods such as October and February is critical to minimizing injury risks. By strengthening surveillance and fostering a culture of safety, healthcare institutions can ensure better protection for healthcare workers while improving the overall quality of care. A prospective design, anonymous self-reported surveys, or active monitoring systems to record both reported and unreported injuries would be necessary to measure real prevalence. Reducing occupational exposure might be achieved by implementing targeted treatments during peak months, such as October, February, and January, when needlestick injury rates are highest. Increased staff training, reinforcement of safety procedures, the deployment of extra workers during times of high workload, and improved oversight of compliance with sharps safety regulations are some examples of these interventions. NSIs were few, generally unintentional, and focused in high-risk departments. Injuries were probably caused by underreporting and violations of safety procedures. To lower occupational exposure, targeted actions during peak months, improved reporting, safety-engineered devices, and improved training are advised. These results can inform broader strategies for reducing NSI incidence in similar healthcare settings.
